# Therapeutic roles and molecular mechanisms of Cuscutae Semen in reproductive diseases

**DOI:** 10.3389/fimmu.2025.1659342

**Published:** 2025-09-19

**Authors:** Kun Li, Haixia Zheng, Wenjie Hao, Yanni Kong, Qianru Zhou

**Affiliations:** ^1^ School of Pharmacy, Hangzhou Medical College, Hangzhou, Zhejiang, China; ^2^ School of Basic Medical Sciences and Forensic Medicine, Hangzhou Medical College, Hangzhou, Zhejiang, China; ^3^ Center for Reproductive Medicine, Department of Traditional Chinese Medicine, Zhejiang Provincial People’s Hospital (Affiliated People's Hospital), Hangzhou Medical College, Hangzhou, Zhejiang, China

**Keywords:** Cuscutae Semen, herb, reproductive disease, infertility, premature ovarian insufficiency, recurrent spontaneous abortion, polycystic ovary syndrome, hypothalamus-pituitary-gonadal axis

## Abstract

Cuscutae Semen (CS), one of the herbs in traditional Chinese medicine, has been recorded for a long time and contains various bioactive compounds, including flavonoids, phenolic acids, lignans, and polysaccharides. These compounds contribute to the medicinal effects on reproductive health. This review examines the therapeutic applications of CS in male and female reproductive diseases, its toxicity, and potential herb-drug interactions. This study focused on the medicinal roles and possible molecular mechanisms of CS in the treatment of reproductive diseases, such as male and female infertility, premature ovarian insufficiency, recurrent spontaneous abortion, and polycystic ovary syndrome. This review examines the mechanisms by which CS regulates the hypothalamic-pituitary-gonadal axis, hormone levels, and key molecules and signaling pathways crucial for reproductive health. By elucidating the potential mechanisms of CS action in reproductive endocrine disorders, this study aimed to provide a scientific basis and evidence for its clinical application in these conditions. These findings provide insights into the molecular mechanisms by which CS has significant potential for treating a range of reproductive disorders.

## 1 Introduction

*Cuscuta chinensis* (*C. chinensis*) Lam., a member of the Convolvulaceae family, is widely used in traditional Chinese Medicine (TCM) to treat various diseases. As a tonic Chinese medicine, Cuscutae Semen (CS; Tusizi in Chinese) is known for its effects on nourishing the kidneys, replenishing essence, consolidating essence, reducing urine, calming the fetus, and improving eyesight (1). Furthermore, current research shows that CS also improves other diseases, such as Alzheimer’s disease (2), osteoporosis (3), kidney yang deficiency syndrome (4), diabetic chronic kidney disease (5), and aging (6). It may exert its therapeutic effects by regulating delayed aging and the immune system (7) and inhibiting oxidation (8).

**Figure 1:**
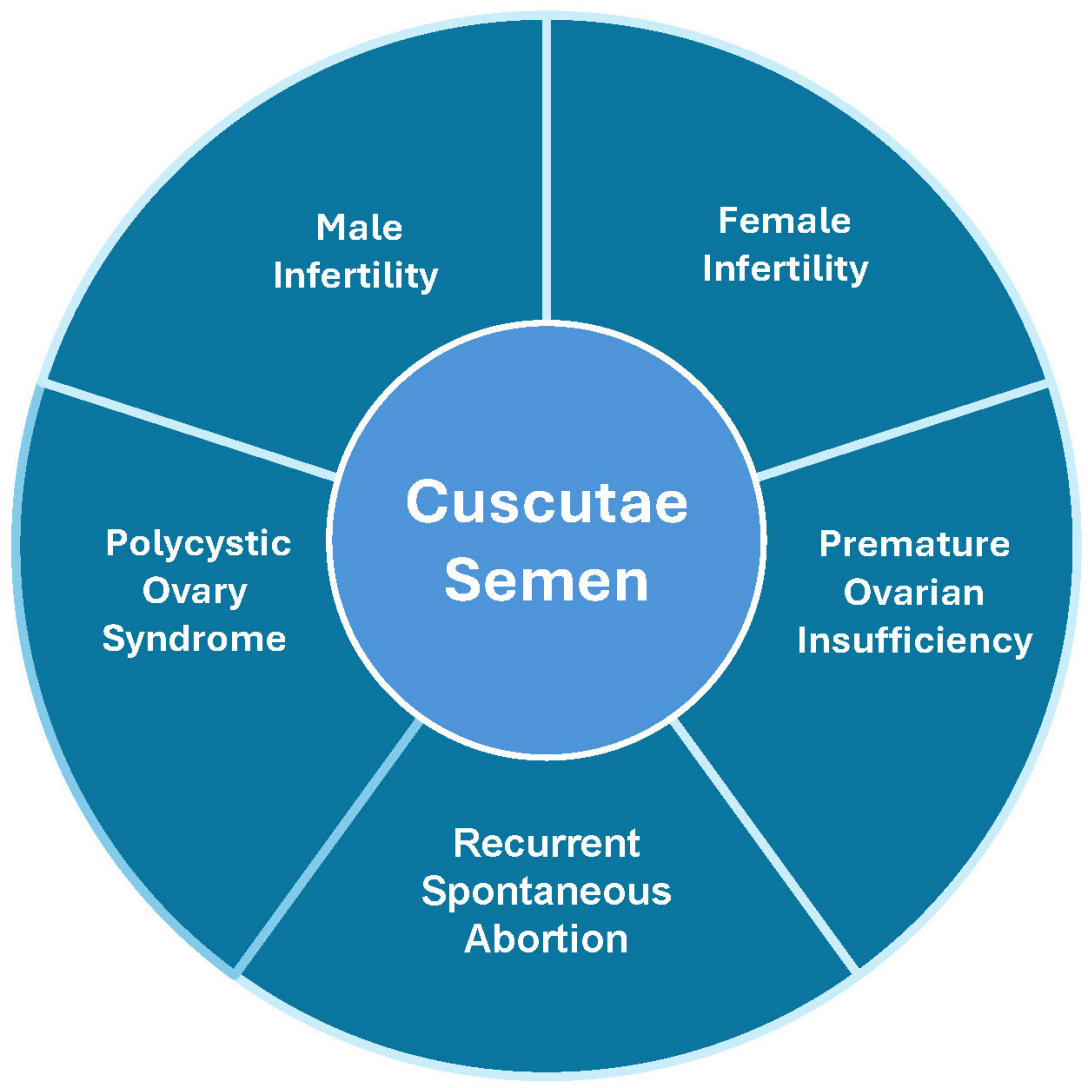
Main reproductive disorders treated with CS.

Furthermore, CS has been used to treat both male and female reproductive disorders (**Figure 1**). It is used to enhance male reproductive function (9) and treat female infertility, and was later explored to treat a series of other reproductive diseases, such as premature ovarian failure (10). Several studies have reported its application in the treatment of reproductive endocrine diseases (11–14). In modern medicinal research, the chemical composition and potential drug activity of CS have become hot research topics (1). CS’s traditional roles in ‘kidney nourishment’ and ‘essence replenishment’ are validated as mechanisms involving hypothalamus-pituitary-gonadal (HPG) axis regulation (9, 15), antioxidant protection of reproductive cells (16, 17), and modulation of hormone receptors (ERα/AR) (18, 19). For example, ‘fetal stabilization’ correlates with flavonoid-mediated inhibition of trophoblast apoptosis via MAPK pathways (20, 21). However, the comprehensive and potential target molecules and signaling pathways of the underlying mechanisms from the perspective of molecular biology in modern medicine are not fully understood, although an array of efficacy studies of CS application in the treatment of reproductive endocrine diseases has been well documented. Therefore, this review attempts to bridge the gap between traditional knowledge and modern science, focusing on the treatment of reproductive endocrine diseases and the signaling pathways underlying the mechanisms of CS targets in the reproductive endocrine process. This study further clarifies the specific mechanism of action of Cuscutae and provides a scientific and sufficient theoretical basis for its clinical application in reproductive endocrine diseases.

## 2 The origin of CS for disease therapy

CS has been utilized as an herbal remedy, as documented in numerous classical studies of Chinese traditional medicine. CS, classified as a top-grade herb, was initially mentioned in *Shennong’s Herbal Classic*, the earliest Chinese pharmacological work published during the Han Dynasty, and was considered a seminal text in TCM. *Shennong’s Herbal Classic* enumerates Cuscutae and documents its primary medicinal effects, including tonifying the liver and kidneys, enhancing visual acuity, promoting fetal stability, and improving sperm quality (11, 16). The medicinal properties of *Cuscuta australis (C. australis)* were further elucidated in *Lei Gong processed medicinal solutions*. The compatibility of Cuscutae with Astragalus terrestris (i.e., Astragalus membranaceus) was addressed in *the Collected Works of Materia Medica*. The role of CS in kidney yang and its relationship with reproductive essence were expounded in the *Classified Canon*. The application of *C. australis* in the treatment of infertility was noted in *The Secret Records of the Stone Chamber*. The use of *C. australis* in the treatment of female-related gynecological disorders has been discussed in the *Golden Mirror of Medicine*. The treatment of infertility was examined, and the use of *C. australis* was referenced in *Medical Prescriptions*. The application of *C. australis* was mentioned in *The Outline of Jiyang*, which discussed the treatment method involving *Cuscutae chinensis*, as well as the relationship between female infertility and menstrual disorders, and was explored in the medical prescriptions, *The Complete Collection in a Plate of Pearls*. These continuously experienced applications suggest that CS has evident efficacy in disease treatment.

## 3 Major Bioactive Compounds of CS

**Figure 2:**
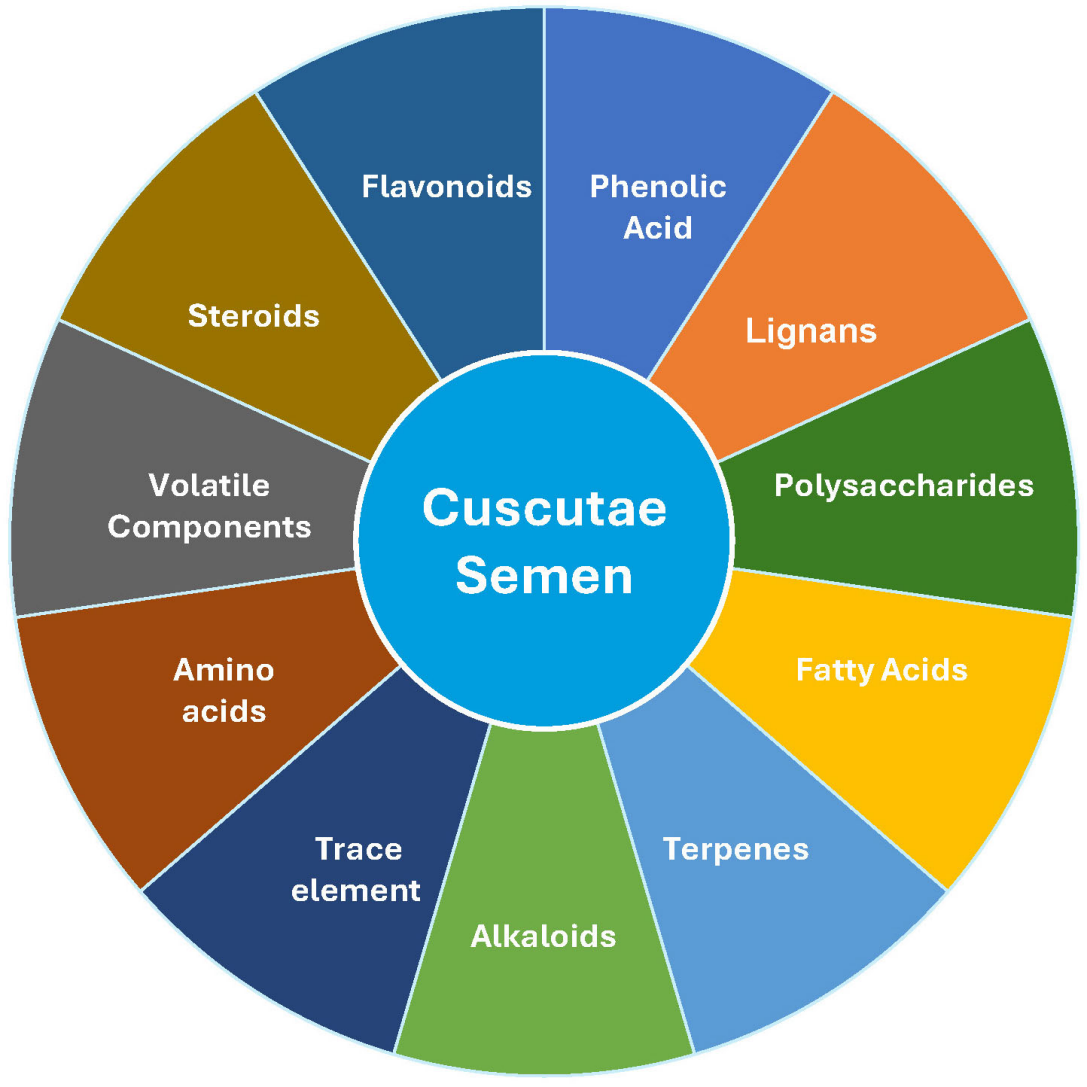
Predominant bioactive compounds in CS.

CS contains a variety of bioactive compounds (**Figure 2**) that contribute to its medicinal properties (1). With the advancement of modern investigation methods and technologies, lignans such as sesamin (22) and polysaccharides, primarily composed of mannose, have been isolated, detected, and characterized (9). These compounds have various biological effects. CS is rich in secondary metabolites, including flavonoids, chlorogenic acids, and lignans (23, 24). UPLC-MS analysis identified 45 predominant phenolic compounds (23). Polysaccharides derived from CS exhibit kidney-nourishing and antioxidant properties (3). Chromatographic techniques have been employed to analyze and quantify their components, including flavonoids (25, 26) and sesamin (22). The total flavonoid content varies among species (27). Recent evidence has shown that substances in CS, including kaempferol, quercetin, apigenin, hyperin, astragalin, and quercitrin, have potential estrogenic effects (28). Additionally, different CS processes lead to different efficacious components (29). These findings provide a scientific basis for the medicinal applications of CS.

### 3.1 Flavonoids

Approximately 40 primary flavonoids have been identified in CS (1). Key flavonoids include quercitrin, quercetin, hyperoside, kaempferol, and astragalin (23, 26). Flavonoids are characterized by a C6-C3-C6 structure derived from a parent nucleus, which influences their chemical properties. Most flavonoids form glycoside compounds when combined with sugars, and exhibit diverse medicinal activities. Research on flavonoids in CS began earlier, and these compounds are among the most extensively studied active ingredients (27).

### 3.2 Phenolic Acids

More than 20 different phenolic acids have been identified in CS, with phenolic acids being the main components of *C. australis* (1). These compounds are characterized by multiple phenolic hydroxyl groups attached to the benzene ring. Phenolic acids are medicinally active ingredients that act as anti-inflammatory, antibacterial, and immune-boosting agents.

### 3.3 Lignans

More than 20 different lignans have been identified in C. *chinensis* (1). Lignans are natural compounds produced through the oxidation and polymerization of phenylpropanoids and are characterized by a core structure containing one or more C6-C3 fundamental units. It is one of the primary bioactive components of *C. chinensis* (23, 24).

### 3.4 Polysaccharides

CS contains several neutral heteropolysaccharides, such as galactose, glucose, mannose, rhamnose, arabinose, and xylose. Glycosidic bonds are linked to complex molecules. One notable polysaccharide derived from *C. chinensis* is the Cuscutae polysaccharide, which demonstrates a range of pharmacological benefits including antitumor and anti-aging properties, immune system modulation, blood glucose reduction, and lipid regulation (1, 30).

### 3.5 Fatty acids

Fatty acids are organic compounds composed of carbon, hydrogen, and oxygen, featuring extended carbon chains and carboxyl groups that are essential for biological functions. The fatty acid structure plays a crucial role in various biological processes.

### 3.6 Steroids

Steroids are organic compounds characterized by a fundamental structure consisting of a cyclopentane polyhydrophenanthrene nucleus. Among the four constituent rings, the steroid core exhibited various ring fusion patterns. The hydroxyl groups at the C-3 position often form chemical bonds with sugar molecules, resulting in compounds classified as glycosides.

### 3.7 Terpenes

Terpenoids are compounds derived from mevalonate, which contains diene units (C5 units) as fundamental structural components. The molecular framework of terpenoids is predominantly chain-like or cyclic, incorporating various functional groups, including alcohols, aldehydes, ketones, carboxylic acids, and esters.

### 3.8 Alkaloids

Alkaloids are naturally occurring nitrogen-containing compounds with basic properties. These compounds typically have complex ring structures and exert significant physiological effects. Alkaloids are the key active components of Cuscutae.

### 3.9 Trace elements

Research has revealed the presence of essential trace elements in Cuscutae, including Mn, Cu, Mn, Fe, Zn, P, and Se (31), with Mn and Zn being higher in dodder. Zn is also important for sperm motility.

### 3.10 Amino acids

CS contains various amino acids (32), including seven essential amino acids required by the human body: valine, threonine, isoleucine, phenylalanine, methionine, lysine, and leucine. In addition, two semi-essential amino acids, histidine and arginine, have been identified. Arginine is essential for spermatogenesis and improves sperm motility and concentration (33).

### 3.11 Volatile components

Volatile components are among the primary constituents of CS, a seed-based medicinal material rich in oil.

Diverse bioactive compounds in CS have multidimensional medicinal functions. Additionally, CS from C. *chinensis* and C. *australis* exhibit significant differences in their phenolic profiles, with hyperoside being predominant in C. *chinensis*, and kaempferol and astragalin in *C. australis* (24). However, recent studies have indicated that the seeds of *C. australis* the main medicinal product of *C. australis*, exhibit efficacy similar to that of C. chinensis (34), which belongs to the same subgenus and shares similar chemical composition and content, although earlier research primarily focused on *C. chinensis* as the origin of the dodder.

It is important to note that the major bioactive compounds of Cuscuta species can exhibit variability depending on several factors, including the specific species, the host plant it parasitizes, the geographical location where it grows, and prevailing environmental conditions. This inherent variability underscores the necessity of carefully specifying the Cuscuta species and its origin in both research investigations and therapeutic applications to ensure the consistency and reproducibility of results.

### 3.12 Material basis for medicinal effects

The bioactive compounds in CS exhibit therapeutic effects. Flavonoids are the primary material for treating premature ovarian insufficiency (POI) and recurrent spontaneous abortion (RSA) by modulating PI3K/AKT and inflammatory pathways (10, 17, 20, 28). Kaempferol is the primary component of the Wuzi Yanzong Pill for Asthenozoospermia, including CS. Polysaccharides enhance sperm quality and testosterone levels in infertile males, likely through their antioxidant and kidney-nourishing effects (9, 16, 35). Lignans (e.g., sesamin) and phenolic acids synergistically regulate hormone receptors in reproductive disorders (18, 22, 24). Therefore, these bioactive compounds provide the material basis for the medicinal efficacy of CS in reproductive health therapy.

## 4 Reproduction diseases treated by CS

### 4.1 Male and female infertility

CS, particularly *C. chinensis, is* often incorporated into compound formulations and has been widely utilized for its potential benefits in the treatment of male and female infertility. The Jiawei Wuzi Yanzong Pill formulation combines Cuscutae with other herbs, such as wolfberry and raspberry, to nourish the liver and kidneys, replenish essence and blood, and address male infertility (25, 36). CS is the most commonly used herb for the treatment of female infertility (14). Jiajian Congrong Cuscutae Pills are effective for treating menstrual disorders (37), endometrial hyperplasia (38), and cystic ovarian syndrome (39). Jiajian Shoutai Pills often combine Cuscutae with other tonics to strengthen the liver and kidneys, invigorate the spleen, and nourish the blood, making them effective for conditions such as fetal loss and miscarriage (40).

Recent evidence has provided insights into the efficacy and mechanism of CS in treating infertility (41–43). Research has shown that CS can enhance sperm motility and membrane function *in vitro* and modulate the PPAR signaling pathway (44). indicating benefits for male fertility treatment (42, 45). In animal models, it has been shown to enhance the reproductive ability of rats with kidney yang deficiency (18), suggesting a therapeutic effect on male infertility. CS has been associated with improvements in gynecological health, particularly in conditions associated with kidney deficiency. Its use in the treatment of infertility and menstrual disorders has been documented (41, 43); however, more rigorous scientific studies are required to validate these effects.

The action of CS in treating infertility includes nourishing the kidneys, regulating hormones (35, 46–48), antioxidant properties (17, 49), and improving ovarian function (14). CS is primarily recognized in TCM for its ability to nourish the kidneys, which is crucial for reproductive health. Studies have shown that CS can improve sperm quality by enhancing sperm count, motility, and morphology (35, 48). It is believed that CS can address various deficiencies, including kidney yin and yang deficiency, which can lead to male reproductive issues, such as low sperm count and decreased sperm motility (oligoasthenospermia). In women, CS is thought to regulate menstrual cycles and enhance ovarian function, thereby improving the uterine environment for implantation. It has been used to treat menstrual irregularities, endometrial hyperplasia, and premature ovarian failure (POF).

### 4.2 POI

CS has beneficial effects on POI (50). Specifically, its active components, particularly flavonoids such as quercetin, show promise in treating POI by modulating key signaling pathways and improving ovarian function. For instance, these compounds have been found to inhibit POI progression by modulating the PI3K-AKT signaling pathway. Flavonoids found in CS have been shown to enhance the expression of estrogen receptors in the hippocampus, hypothalamus, and pituitary gland (51) as well as luteinizing hormone (LH) receptors in the ovaries of stressed rats, suggesting a potential mechanism by which CS may support ovarian function (10). TCM formulations, such as the Bushen Culuan Decoction, have also shown promise in ameliorating POI by activating antioxidant pathways, which may help protect ovarian function (52). Furthermore, CS has been shown to improve ovulation disorders associated with kidney deficiency by promoting follicular development and increasing the number of secondary follicles (53).

CS and its active components, particularly flavonoids such as quercetin, show promise in the treatment of POI by modulating key signaling pathways and improving ovarian function (10, 17, 54). Compounds in CS, particularly quercetin and other flavonoids, have been found to inhibit POI progression by modulating the PI3K-AKT signaling pathway (10). This pathway is crucial for cell survival and growth, and its modulation may improve ovarian function and hormone levels. Other compounds, such as epicatechin, have shown potential for treating POI via the PI3K/AKT/Nrf2 pathway (52), further supporting the idea that various herbal treatments can target multiple mechanisms involved in ovarian health. TCM formulations containing CS have demonstrated efficacy in improving ovarian function and hormone levels in POI models. These formulations may affect multiple signaling pathways involved in POI, including the PI3K/Akt/mTOR Pathways (19), thereby reducing follicular atresia and enhancing ovarian reserve. CS exhibits a range of medicinal activities, including antioxidant (17, 49), hepatoprotective (55), and modulation of arachidonic acid, glycerophospholipid, and linoleic acid metabolism, as well as steroid hormone biosynthesis (56), which may contribute to its therapeutic potential in the treatment of POI. Other herbal medicines, such as Bushen Huoxue, also act on POI by regulating the TGF-β1 and Smad2/3 signaling pathways (52). CS combined with acupuncture may be beneficial in treating POI, including normalizing menstrual cycles and improving hormone levels, as meta-analyses suggest that this combination may be more effective than hormone replacement therapy in managing symptoms associated with POI (50). However, further high-quality clinical trials are necessary to confirm these findings and to establish the efficacy and safety of herbal treatments for POI.

### 4.3 RSA

CS has shown promising effects in the treatment of RSA and in enhancing overall reproductive health (13). Research indicates that flavonoids in CS play a key role in this effect by regulating inflammatory pathways and promoting trophoblast cell invasion, which are crucial for maintaining a healthy pregnancy (20). Studies have demonstrated that flavonoids in CS effectively modulate inflammatory responses (57, 58). This regulation is essential for creating a favorable environment for implantation and fetal development, thereby reducing the risk of spontaneous abortion. In animal models of abortion, CS influences the expression of important proteins, such as Fas, PCNA, and HB-EGF (59). These proteins are involved in cell survival, proliferation, and trophoblast function, suggesting that CS may help maintain pregnancy by enhancing trophoblast activity. CS also exhibits anti-osteoporosis effects in ovariectomized mice (3), indicating its potential to improve bone health under postmenopausal conditions. Flavonoids from CS can ameliorate ovarian endocrine dysfunction, particularly in stressed female rats. CS may help restore hormonal balance and improve ovarian function by modulating hormone receptor expression (60, 61), thus further supporting reproductive health.

### 4.4 Polycystic ovary syndrome (PCOS)

A recent study demonstrated the effects of CS in an established PCOS rat model (62). Another study demonstrated that CS, when combined with Salvia, regulates ten core genes involved in PCOS treatment, including IL6, AKT1, VEGFA, TP53, TNF, MAPK1, JUN, EGF, CASP3, and EGFR (63). The experimental animals exhibited abnormalities in the HPG axis, and the administration of varying doses of total flavonoids from CS to PCOS model rats resulted in significant improvements in pathological manifestations. Notably, serum E2 levels were significantly increased, whereas the ovarian index was reduced, indicating a positive effect on ovarian function. CS may play a role in regulating hormonal fluctuations associated with the menstrual cycle because the total flavonoids from CS were found to enhance the concentration of β-endorphin (β-EP) during the luteal phase, which subsequently decreased rapidly (15). CS have been shown to upregulate serum E2 levels and enhance the function of human chorionic gonadotropin (hCG) (64). This evidence suggests that CS may be involved in PCOS pathogenesis. However, it is important to note that CS did not have a significant effect on FSH receptor (FSHR) expression in ovaries (60). Collectively, CS may be beneficial for the treatment of polycystic ovary syndrome by improving hormonal balance and ovarian function. Further research is needed to fully understand the mechanisms and clinical implications of CS in PCOS management.

### 4.5 Clinical evidence on CS treatments for reproductive disorders

Some clinical evidence involving CS is emerging, while most research on CS is preclinical in nature. Meta-analyses support CS-containing formulations for reproductive disorders, although treatments using CS alone have been less investigated. A recent meta-analysis systematically evaluated the effectiveness of CS-containing Wuzi Yanzong pills in the treatment of oligoasthenospermia in male infertility. The analysis included a total of 16 studies involving 1960 patients, demonstrating that the Wuzi Yanzong pill alone is superior to Western medicine in improving sperm density, sperm motility, conjugal pregnancy rate, and total effective rate (65). The effect of CS contributed to the main role of the Wuzi Yanzong pill because the predominant composites in the formulation were derived from CS, as detected by modern technology (25).

Moreover, a Taiwanese cohort study identified CS as the most commonly prescribed herb for addressing female infertility (41), and a meta-analysis reported that CS were the most frequently observed cases with kidney deficiency acts as a “reinforcing yang herb” to strengthen the liver and kidney, and indicated that herbal medicine significantly boosted pregnancy rates when compared to placebo treatments (43). Another meta-analysis suggested that CS combined with acupuncture may be more effective than hormone replacement therapy for managing POI symptoms (50). Moreover, CS, as one of the important Chinese herbs, was discussed in a meta-analysis to have potentially beneficial effects on the perinatal outcomes of pregnant women in clinical applications (51).

In addition to the meta-analysis, a clinical trial involving a CS-containing formula, designed as a multicenter, randomized, double-anonymized, placebo-controlled study, has been conducted as a therapeutic option for reviving residual follicles in POI, and the study presented a promising strategy to delay ovarian decline and produce significant clinical evidence (66).

However, clinical trials on single CS therapy are lacking, except for clinical trials on formulations containing CS. Meanwhile, the methodological quality of the RCTs may have been poor, although the risk of bias in the included RCTs was generally low. Therefore, more high-quality clinical trials are required to confirm these findings.

## 5 Molecular mechanisms by which CS play the medicinal roles in treating reproductive diseases

### 5.1 Effects of CS on reproductive endocrine

**Figure 3:**
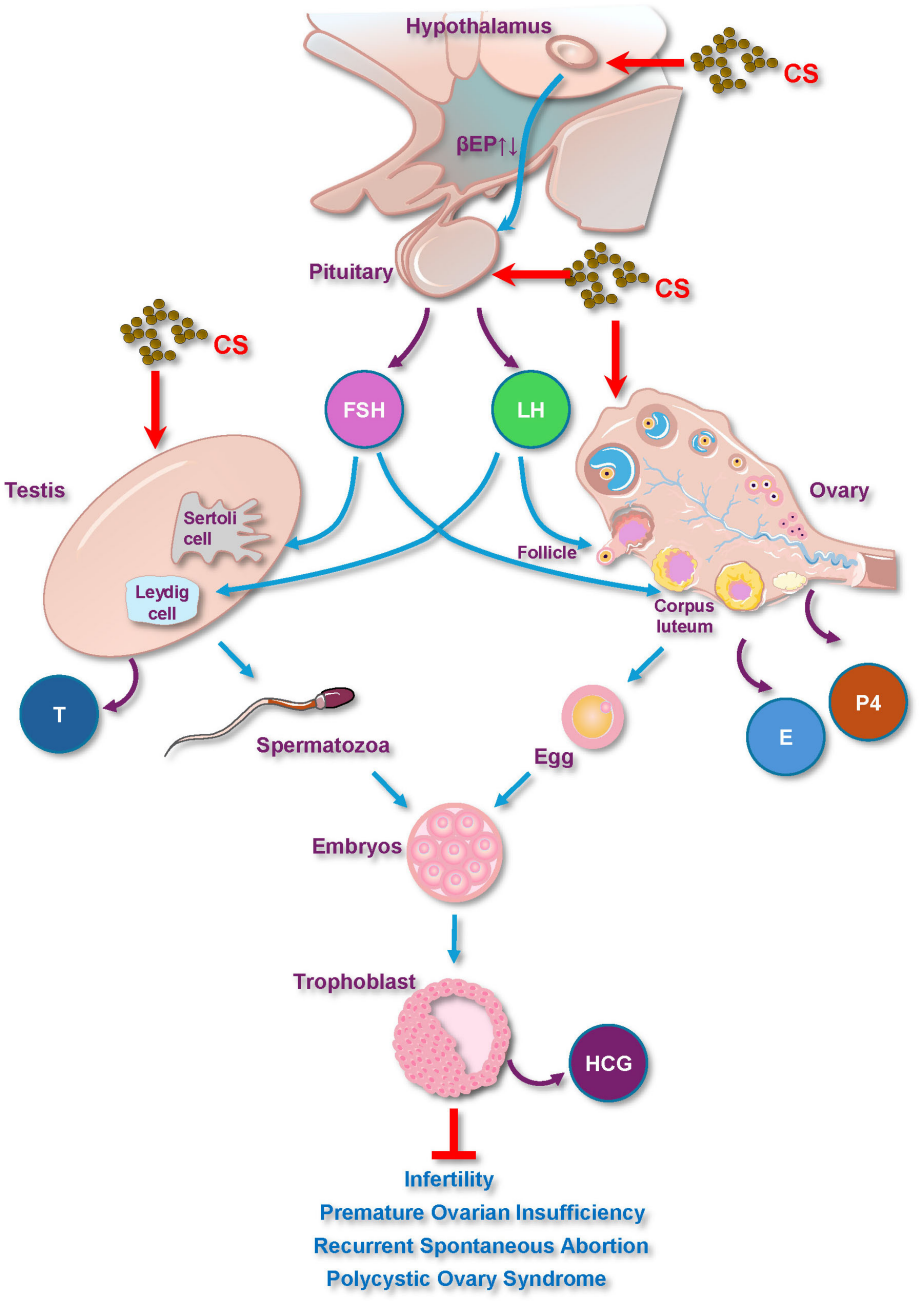
CS plays a medicinal role in reproductive diseases via the hypothalamus-pituitary-gonadal axis and hormones. E, Estrogen; FSH, Follicle-Stimulating Hormone; LH, Luteinizing Hormone; P4, Progesterone; T, Testosterone; HCG, Human Chorionic Gonadotropin.

CS has garnered attention for its medicinal effects on the reproductive system, particularly through its influence on the hypothalamus-pituitary-gonadal (HPG) axis (60). This intricate neuroendocrine system, comprising the hypothalamus, pituitary gland, and gonads (ovaries or testes), serves as the primary regulatory center for reproductive hormone production and secretion, and plays a vital role in controlling reproductive functions (**Figure 3**).

#### 5.1.1 HPG axis

CS modulates the HPG axis to regulate reproductive health. Specifically, studies have shown that CS total flavonoids promote the synthesis and secretion of gonadotropin-releasing hormone (GnRH) from the hypothalamus (67).

#### 5.1.2 Hypothalamus

The hypothalamus plays a crucial role in regulating endocrine activity by secreting GnRH, which acts on the pituitary gland to stimulate the synthesis of prolactin and gonadotropic hormones (68). Under stressful conditions, corticotropin-releasing factor (CRF) inhibits GnRH synthesis, leading to reproductive dysfunction. As mentioned above, flavonoids from CS decrease the content of beta-EP in the hypothalamus (15).

#### 5.1.3 Pituitary

The pituitary gland synthesizes and secretes FSH and LH, which are essential for steroid production in the gonads. Furthermore, flavonoids from CS increase LH levels in the anterior pituitaries (15). Another study showed that the modified Congrong Tusizi pill containing CS also enhances the function of the HPG axis by improving the pituitary response to GnRH from the upstream hypothalamus (69). However, the mechanism by which CS directly influences the pituitary gland requires further study.

#### 5.1.4 Gonads

In males, LH promotes the differentiation of Leydig cells, leading to androgen production in the testes, whereas FSH stimulates spermatogenesis. Evidence also shows that CS increases testicular, epididymal, and pituitary weights and stimulates testosterone and LH secretion in rats (16, 18, 70).

In females, hormones and their receptors are critical for follicular growth. Upstream signals triggered by total flavonoids from CS can lead to a significant increase in E2, LH, and hCG levels as well as changes in FSHR receptors (59, 60), and can promote the ovarian response during the maturation of dominant follicles and increased proliferation of follicular granulosa cells (69).

### 5.2 Involvement of CS in diverse signaling pathways

**Figure 4:**
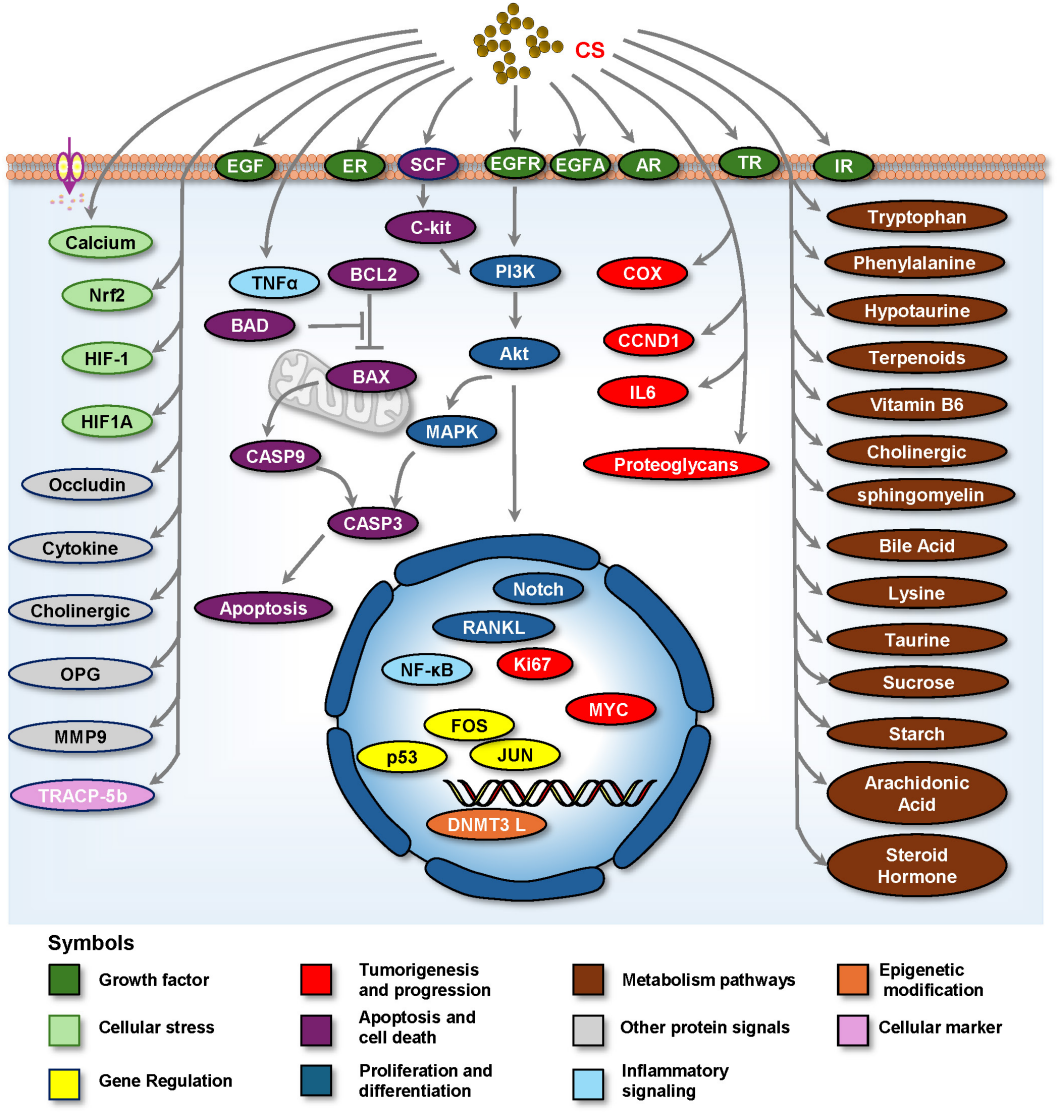
Cell signaling by which CS plays a medicinal role in treating reproductive diseases. Abbreviation: AR, Androgen Receptor; BAD, Bcl-2-Associated Death Promoter; BAX, Bcl-2-Associated X Protein; BCL2, B Cell Lymphoma 2; CASP3, Caspase-3; CCND1, Cyclin D1; COX, Cyclooxygenase; DNMT3L, DNA Methyltransferase 3-Like; EGF, Epidermal Growth Factor; EGFR, Epidermal Growth Factor Receptor; ER, Estrogen receptor; FOS, FBJ murine osteosarcoma viral oncogene homolog; HIF-1, Hypoxia-Inducible Factor-1; HIF1A, Hypoxia-Inducible Factor 1-Alpha; IL6, Interleukin-6; IR, Insulin Receptor; JUN, Jun oncogene; MAPK, Mitogen-Activated Protein Kinase; MMP9, Matrix Metalloproteinase 9; MYC, myelocytomatosis oncogene; NF-κB, Nuclear Factor-Kappa B; Nrf2, Nuclear Factor Erythroid 2-Related Factor 2; OPG, Osteoprotegerin; PI3K, Phosphatidylinositol 3-Kinase; TNF-α, RANKL, Receptor Activator of Nuclear Factor-κB ligand; Tumor Necrosis Factor-Alpha; TP53, TR, Thyroid hormone receptor; Tumor Protein P53; TRACP-5b, Tartrate-resistant Acid Phosphatase 5b; VEGFA, Vascular Endothelial Growth Factor A; TRACP-5b, Tartrate-resistant Acid Phosphatase 5b; VEGFA, Vascular Endothelial Growth Factor A.

CS exerts various medicinal effects on the reproductive system by regulating specific molecules and signaling pathways. The different pathways are illustrated in **Figure 4** and summarized in **Table 1**. This section outlines the key targeted molecules and signaling pathways influenced by CS, highlighting its potential mechanisms of action in reproductive health.

**Table 1:** Signaling pathways, molecules, and their functions involved in the pharmacological roles of CS.

Signaling pathways	Molecules	Functions	References
Growth Factor Signaling	EGFR and EGF	Epidermal growth factor receptor signaling	(17, 59, 71, 72)
	VEGFA	Vascular endothelial growth factor signaling	(71, 72)
	Insulin	Insulin receptor signaling	(73)
	AR	Androgen signaling	(18)
	Estrogen Receptor	Estrogen receptor signaling	(19, 74)
	Thyroid Hormone Receptor	Thyroid hormone receptor signaling	(47)
Proliferation and Differentiation Signaling	PI3K/Akt and MAPK	Cell growth and survival	(10, 17, 20, 21, 45, 48, 54, 70, 72, 76)
	Notch	Cell fate determination	(20)
Cellular Stress and Survival Signaling	Calcium	Calcium signaling	(76)
	Nrf2	Nrf2 pathway activation, antioxidant response signaling	(17, 52, 78)
	HIF-1 and HIF1A	HIF1A hypoxia response signaling	(13)
Inflammatory Signaling	NF-κB	Inflammatory response and immune regulation	(58, 79)
	TNF-α	Tumor necrosis factor signaling	(13, 57, 58, 79)
	Oxidative Stress Molecules	Pathway response to oxidative stress	(16, 17, 35, 49, 52, 54, 55, 78, 79)
Apoptosis and Cell Death Regulation	BAD, BAX, Cleaved CASP3, CASP3	Inducers of apoptosis and cell death regulation	(80)
	Cleaved CASP3,	Apoptosis and cell death regulation	(48, 54, 58)
	Bax	Regulation of pro-apoptotic protein Bax	(54)
	BCL2	Anti-apoptotic protein	(46)
	SCF/c-kit-PI3K-Bcl2	Survival signaling pathway	(48)
Tumorigenesis and Progression	MYC	Cell cycle regulation	(74)
	Proteoglycans	Tumor biology	(13)
	COX	Cancer and inflammation	(57, 58, 79)
	IL6	Cytokine involved in cancer progression	(72)
	CCND1	Cell cycle regulation	(72)
	Ki67	Proliferation marker in cancer	(42)
Metabolism Pathways	Tryptophan	Pathways involving tryptophan	(4, 82)
	Phenylalanine	Pathways involving phenylalanine	(4, 82)
	Lysine and Vitamin B6	Amino acid degradation and vitamin metabolism	(4)
	Taurine and Hypotaurine	Taurine metabolism	(4)
	Arachidonic Acid	Inflammatory processes	(4, 83)
	Sphingomyelin	Lipid metabolism related to cellular membranes	(4)
	Starch and Sucrose	Carbohydrate breakdown	(4)
	Glucose Transporter	Regulation of glucose uptake	(81)
	Primary Bile Acid	Synthesis of bile acids	(4)
	Steroid Hormone	Synthesis of steroid hormones	(4)
	Terpenoid Backbone	Synthesis of terpenoids	(3, 4)
Gene Regulation	TP53	Tumor suppressor and transcription factor	(19, 71)
	FOS	Immediate early gene and transcription factor	(3, 84)
	JUN	Transcriptional regulation	(26, 48, 88)
Epigenetic Modification	DNMT3 L	Epigenetic regulation	(47, 73)
Cellular Markers and Indicators	TRACP-5b	Marker for bone resorption	(3, 86, 87)
Cellular Functions and Modulation	Endothelial Function Modulation Proteins	Regulation of endothelial cell function	(14, 72)
Other potential signals	MMP9	Tissue remodeling	(20)
	Occludin	Cell adhesion	(88)
	Osteoprotegerin (OPG)	Metabolism	(86–88)
	Receptor Activator of Nuclear Factor-κB (RANKL)	Bone remodeling	(3, 86–88)
	Cytokine	Immune modulation	(7, 14, 79)
	Cholinergic	Immune modulation	(8, 89)

AR, Androgen Receptor; BAD, Bcl-2-Associated Death Promoter; BAX, Bcl-2-Associated X Protein; BCL2, B Cell Lymphoma 2; CASP3, Caspase-3; CCND1, Cyclin D1; COX, Cyclooxygenase; DNMT3L, DNA Methyltransferase 3-Like; EGF, Epidermal Growth Factor; EGFR, Epidermal Growth Factor Receptor; FOS, FBJ murine osteosarcoma viral oncogene homolog; HIF-1, Hypoxia-Inducible Factor-1; HIF1A, Hypoxia-Inducible Factor 1-Alpha; IL6, Interleukin-6; JUN, Jun oncogene; MAPK, Mitogen-Activated Protein Kinase; MMP9, Matrix Metalloproteinase 9; MYC, myelocytomatosis oncogene; NF-κB, Nuclear Factor-Kappa B; Nrf2, Nuclear Factor Erythroid 2-Related Factor 2; PI3K, Phosphatidylinositol 3-Kinase; TNF-α, Tumor Necrosis Factor-Alpha; TP53, Tumor Protein P53; TRACP-5b, Tartrate-resistant Acid Phosphatase 5b; VEGFA, Vascular Endothelial Growth Factor A; TRACP-5b, Tartrate-resistant Acid Phosphatase 5b; VEGFA, Vascular Endothelial Growth Factor A.

#### 5.2.1 Growth factor signaling

CS affects growth factor signaling, which is vital for cell proliferation and differentiation. There are many different signaling molecules, including EGFR, EGF (17, 59, 71, 72), VEGF (71, 72), insulin receptor (73), androgen receptor (AR) (18), estrogen receptor (19, 74), and thyroid hormone receptor (19, 74). CS can promote the development and function of reproductive tissues by enhancing the activity of growth factors.

#### 5.2.2 Proliferation and survival signaling

CS may enhance the activation of this pathway, promoting cell proliferation and survival in reproductive tissues via the PI3K/AKT Pathway, which is involved in cell survival, growth, metabolism (10, 17, 48, 54, 70), and cancer (75). CS may modulate the mitogen-activated protein kinase (MAPK) pathway, which plays a significant role in cell differentiation and response to growth factors (17, 20, 21, 45, 54, 72, 76), thereby influencing cellular responses in the reproductive system. Furthermore, CS activates the Notch signaling pathway (20), which is essential for cell fate determination and differentiation. This pathway may be involved in ovarian function and follicular development. Furthermore, CS flavonoids activate the Notch/AKT/MAPK signaling pathway, which plays a role in the treatment of ovarian endocrine and reproductive disorders (20). Moreover, CS affects several key signaling pathways that are critical for cellular responses to external stimuli, stress, and survival signals. The relieved stress may be involved in protecting testicular tissues (77). These include calcium (76), Nrf2 (17, 52, 78), HIF-1, and HIF1A (13).

#### 5.2.3 Inflammatory signaling

CS may modulate inflammatory responses and immunity via inflammatory signaling pathways, including NF-κB Signaling (58, 79) and TNF-α signaling (13, 57, 58, 79), which are critical for maintaining reproductive health. By regulating the inflammatory response, CS can protect reproductive tissues from damage and promote healing.

Furthermore, CS affects oxidative stress, which is closely linked to inflammation. The polysaccharides and flavonoids found in CS exhibit antioxidant properties that protect reproductive cells from oxidative stress (16, 35, 52). Studies have indicated that these compounds can enhance the ability to scavenge free radicals, thereby reducing damage to spermatogenic cells and promoting testosterone secretion (9, 16).

#### 5.2.4 Apoptosis and cell death signaling

CS may regulate apoptosis by modulating pro- and anti-apoptotic proteins. By balancing these proteins, CS can influence cell survival and death in reproductive tissues, potentially protecting cells against stress-induced apoptosis (80). Proteins potentially involved in regulating apoptosis and cell death include pro-apoptotic proteins such as BAD, BAX, Cleaved Caspase 3, and CASP3, as well as those involved in regulating cell death (48). Cleaved Caspase 3 is linked to the regulation of the pro-apoptotic protein Bax (48). The anti-apoptotic protein BCL2 was also noted (54), along with the CSF/c-kit-PI3K-Bcl-2 pathway, which is a survival signaling pathway (46). All of these signaling pathways are closely associated with the physiology and pathology of reproductive endocrinologic systems.

#### 5.2.5 Metabolic pathways

CS is involved in various metabolic pathways that are crucial for cellular energy production and material transformation. Key metabolic processes influenced by CS include carbohydrate breakdown and regulation of glucose uptake, which are essential for cellular energy production. CS may enhance metabolic activity, including starch and sucrose metabolism, as well as glucose transporter regulation (81), thereby supporting the energy demands of reproductive cells.

Amino acid metabolism includes tryptophan metabolism (4, 82), phenylalanine metabolism (4, 82), lysine degradation, vitamin B6 metabolism (4), and taurine and hypotaurine metabolism (4). CS may influence lipid synthesis and breakdown, which are crucial for maintaining the integrity of the cellular membrane and hormone production. Arachidonic acid metabolism is involved in inflammatory processes (4, 83). Sphingomyelin metabolism is closely linked to lipid metabolism in cellular membranes (4).

#### 5.2.6 Gene regulation

CS has been implicated in the regulation of gene expression through various mechanisms, including the modulation of transcription factors, such as the tumor suppressor p53 (19, 71) and immediate-early gene transcription factors FOS (3, 84) and JUN (26, 48). By influencing these regulatory elements, CS can affect the expression of genes involved in reproductive processes, including hormone synthesis and cellular differentiation.

#### 5.2.7 Epigenetic modifications

Research suggests that CS may play a role in epigenetic regulation, affecting gene expression without altering the DNA sequence, such as DNA methylation regulated by DNMT3L and DNA methyltransferase, which are involved in the regulation of epigenetic modifications (47, 73) and can affect cellular responses in the ovary (85) and reproductive health.

#### 5.2.8 Cellular markers

CS may influence specific cellular markers that are crucial for identifying and classifying cell types in the reproductive system. These markers include proteins, enzymes, and other biomarkers involved in cellular function and differentiation. For example, CS affects Tartrate-resistant Acid Phosphatase 5b (TRACP-5b), a marker of bone resorption (3, 86, 87).

#### 5.2.9 Other potential signals

CS also regulates other signals, including MMP9 in Tissue remodeling (20), Occudin in cell adhesion (42), OPG, and RANKL in bone remodeling (3, 86–88), cytokines (7, 14, 79), and cholinergic signaling in immune modulation (8, 89).

### 5.3 Integration of molecular mechanisms

**Figure 5:**
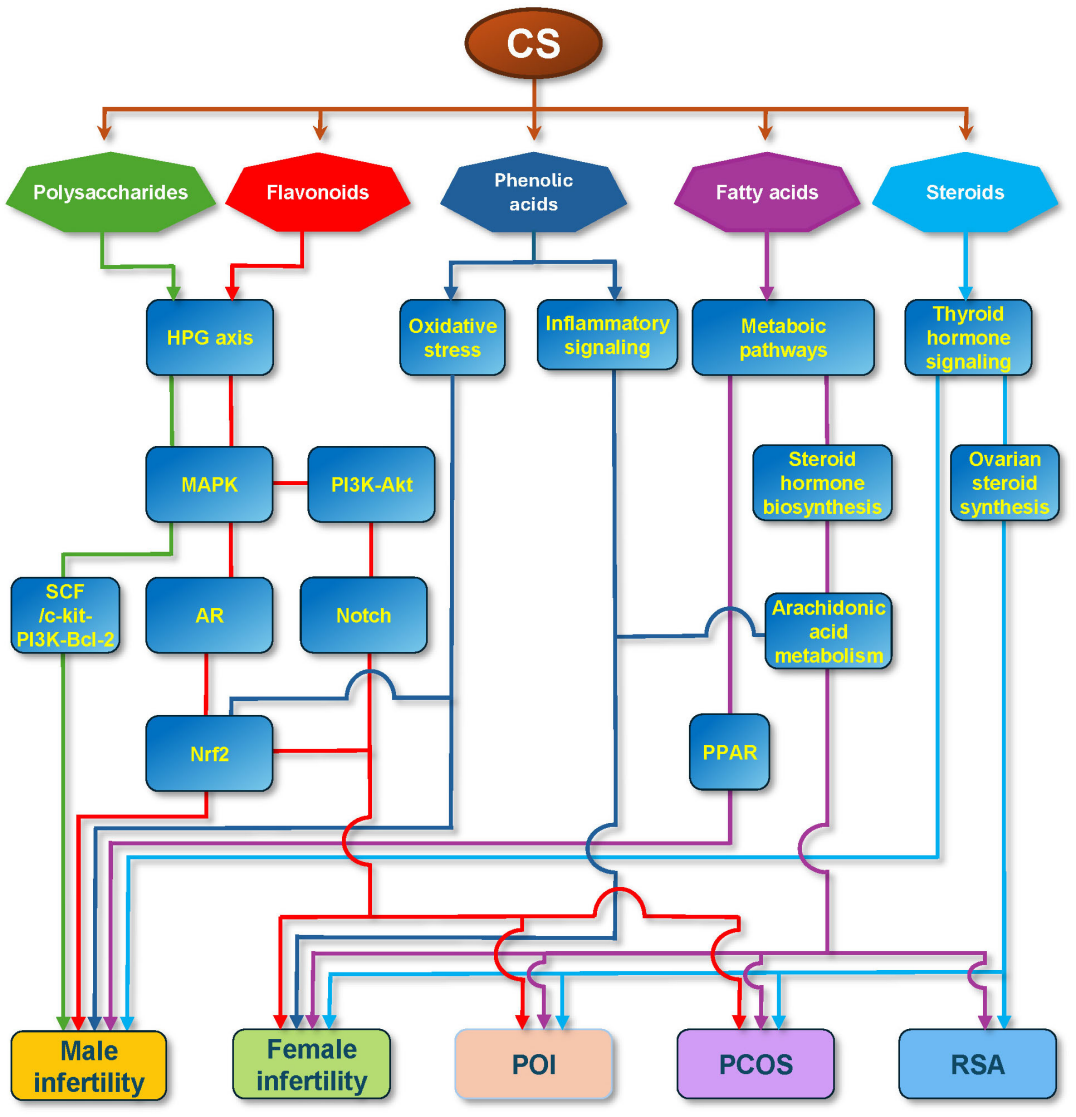
Integration of Molecular Mechanisms of CS Therapy for Reproductive Diseases. The schematic delineates the multifaceted pharmacological profile of CS by illustrating the interconnections among the documented bioactive constituents, underlying molecular mechanisms, and therapeutic efficacies in male infertility, female infertility, primary ovarian insufficiency (POI), polycystic ovary syndrome (PCOS), and recurrent spontaneous abortion (RSA). The figure illustrates that the signaling pathways associated with the five primary chemical classes originate from CS: polysaccharides (green), flavonoids (red), phenolic acids (blue), fatty acids (purple), and steroids (cyan). The lines without arrows represent the parallel relationships between different signal molecules.

Molecular mechanisms of several defined compounds in CS therapy for reproductive diseases have been documented, mainly including five primary chemical classes: polysaccharides, flavonoids, phenolic acids, fatty acids, and steroids. These different components play an important role in reproductive diseases through diverse signaling pathways (integrated in **Figure 5**). There exists convergence in different signaling pathways in the treatment of reproductive diseases by CS. On one hand, different ingredients of CS converge on the same molecules via diverse signaling pathways or the same efficacy. For example, in male infertility, polysaccharides and flavonoids share the same HPG axis and MAPK pathway, and phenolic acids and flavonoids share the same Nrf2 pathway. Between female infertility and POI, RSA, or PCOS, phenolic acids and fatty acids share the arachidonic acid pathway. Between males and females, flavonoids play an important role via the same HPG axis, MAPK, and Nrf2 signaling pathways. Signaling crosstalk between different pathways may occur in relevant cells.

CS influences the reproductive system through complex interactions between targeted molecules and signaling pathways. Such interactions result in a wide range of physiological responses. The effect of CS on the reproductive system is further highlighted by its ability to regulate key proteins or receptors. The intricate mechanisms through which CS exerts its effects underscore its potential as a valuable therapeutic agent for various reproductive disorders.

## 6 Other profiles of CS

### 6.1 Toxicity and safety

The toxicity and safety of CS have been investigated (90). *Cuscuta chinensis* Lam. water extract (CLW) is safe for ICR mice when the dose is lower than 1250 mg/kg (90); the LD_50_ (median lethal dose), reflecting acute toxicity, was over 5000 mg/kg in ICR mice; and subacute toxicity results showed no mouse death or severe toxic reactions. Some indicators changed when different doses were tested in the study: the body weight at doses of 1250, 2500, and 5000 mg/kg CLW in both male and female mice was reduced, and only the feed intake of female mice was reduced; only at the 2500 mg/kg and 5000 mg/kg doses, CLW had very few organ coefficients involving organs such as the brain, liver, lungs, testes (males), and thymus; decreased platelet count, lymphocyte count, and hematocrit and increased neutrophil count; and affected liver functions, increasing aspartate aminotransferase (AST), alanine aminotransferase (ALT), and alkaline phosphatase (ALP), and decreasing albumin, total cholesterol, and triglycerides; only at the 5000 mg/kg dose, daily oral administration of CLW induced mild toxic effects on the liver in both sexes, which may be a matter of concern (90).

CS has low toxicity and is considered safe because its safe dosage is significantly higher than the 6–12 g of dried crude herb recommended daily for humans by the China Pharmacopoeia, although standardized dosing guidelines are not well established. At a dosage of 1250 mg/kg CLW, the safe dosage in mice (90) may be converted to approximately 6.6 grams CLW per day in humans, according to the standard for inter-species dose conversion using the body surface area normalization method. Furthermore, 1250 mg/kg in CLW is 16.2 times (estimated from 6.17% extraction yield) concentrated from the aqueous extract of dried crude herb (90), so 6.6 grams CLW each day in humans is equivalent to 106.9 grams dried crude herb, which is much higher than the dosage recommended by the China Pharmacopoeia, which also suggests that the Pharmacopoeia dose is safe. According to the safe dosage, it is speculated that the adverse effects of CS in humans are very few, although there is limited information on the adverse effects of CS in humans.

### 6.2 Composition consistency

The consistency of the diverse chemical compositions of CS should be considered. CS contains a wide and distinct array of flavonoids, chlorogenic acids, and lignans (23), as demonstrated by multiple analysis methods. Despite this diversity, the consistent presence of key components, such as chlorogenic acid and quercetin, across various samples indicates a higher degree of uniformity in chemical composition and potential therapeutic benefits (88). Notably, chemical profiles vary by species (e.g., *C. chinensis* vs. *C. australis*), host plants, and geographical location (27, 34). Therefore, the standardization of marker compounds is essential. For example, hyperoside, as recommended by the China Pharmacopoeia, is used to distinguish *C. chinensis* from astragalin in *C. australis* (24). For future applications of CS, it is necessary to clarify its origin, develop cultivation protocols to reduce variability, develop different key compound markers for robust quality control and prediction, and conduct clinical RCTs using standardized extracts.

### 6.3 Herb-drug interaction

CS may be involved in herb-drug interactions, including hormonal drugs, antidiabetic drugs, immunosuppressants, and drug metabolism, although most interactions are based on preclinical studies or theoretical mechanisms. 1) The hormonal drug flavonoids from CS can promote testosterone secretion and expression of androgen receptor (9, 18), ER, and progesterone receptor (61) owing to their effects on the HPG Axis. Thus, it may affect the outcomes of hormonal therapies, such as dydrogesterone treatment (64), including other potential oral contraceptives and hormone replacement therapy, although the evidence is limited. 2) Antidiabetic drugs: A study found that the CS extract reduced blood glucose levels in streptozotocin-induced diabetic rats (91), suggesting synergistic effects with hypoglycemic agents. Concurrent use with antidiabetic drugs may potentiate hypoglycemia, increasing the risk of low blood sugar. 3) The immunosuppressant kaempferol from CS attenuates the function of dendritic cells in the immune system (7) and inhibits inflammatory factors such as TNF-α and IL-6 (57). When used in conjunction with immunosuppressants, CS may counteract the immunosuppressive effects of the drugs. 4) The enzymes critical for drug metabolism, flavonoids in CS, may interact with cytochrome P450 (92) and carboxylesterases 2, which affect the metabolism of ester-containing drugs (93). This suggests that CS can alter the function of drug-metabolizing enzymes and plasma levels of drugs metabolized by these enzymes, even altering their efficacy or toxicity. Therefore, CS should be applied at a safe dosage, with caution to avoid potential herb-drug interactions with specific drugs.

## 7 Conclusion and future direction

In summary, *CS* has a wide range of regulatory effects on the reproductive system through its multiple active ingredients, and its mechanism of action involves the comprehensive regulation of reproductive endocrine systems and multiple signaling pathways. The evidence presented in this review consistently points towards flavonoids as one of the primary material bases for CS’s efficacy across various reproductive disorders. However, the contribution of other ingredients to therapeutic efficacy is being studied and discovered. For example, flavonoids modulate the HPG axis, inhibit POI progression via the PI3K-AKT pathway, regulate inflammation in RSA, and improve hormonal balance in PCOS models. Furthermore, this review also discussed the insistence of CS’s traditional applications with the understanding of modern medicine: treating kidney yang deficiency aligns mechanistically with its ability to upregulate testosterone synthesis (18) and rescue oxidative stress in spermatogenic cells (16, 35); menstrual regulation in TCM corresponds to CS-induced follicular development via FSH receptor sensitization (53, 60). This review focuses on the treatment of reproductive endocrine diseases, potential signaling pathways, and the underlying molecular mechanisms of CS action, thereby bridging the gap between traditional knowledge and modern medicine. This study provides a crucial scientific foundation for the application of CS in the treatment of reproductive disorders.

There are still many limitations that need to be addressed in future research. Continued efforts to elucidate the precise molecular mechanisms of action, including the identification of specific cellular targets, receptors, and downstream signaling events involved in the effects of CS on reproductive tissues and endocrine function, are also warranted. Comprehensive pharmacokinetic and pharmacodynamic studies should be performed to fully elucidate the absorption, distribution, metabolism, and excretion of key CS compounds. The potential synergistic or antagonistic effects of the various compounds present in CS, which are crucial for optimizing therapeutic formulations, including herb-drug interactions, should be investigated through both *in vitro* and *in vivo* studies. The translation of encouraging preclinical findings into evidence-based clinical recommendations through rigorous human studies remains a critical next step in harnessing the therapeutic potential of CS in reproductive endocrine health. Furthermore, well-designed and adequately powered randomized controlled clinical trials of CS monotherapy are essential to rigorously evaluate the efficacy and safety of standardized CS extracts or isolated compounds for the treatment of specific reproductive endocrine diseases in humans.

Future studies should explore the optimal dosage, formulation (including the potential for targeted delivery), and duration of CS-based interventions for different reproductive endocrine disorders. Finally, investigating the potential of CS to address specific symptoms and subtypes of reproductive endocrine disorders will contribute to a more refined and personalized approach for its therapeutic application. For CS to be developed into a standardized modern therapeutic, future studies must focus on developing standardized extracts with consistent chemical profiles and predictable efficacy, which is essential for ensuring the safety and reproducibility of clinical outcomes.

## Glossary

AR: Androgen Receptor
BAD: Bcl-2 Antagonist of Cell Death
BAX: Bcl-2-Associated X Protein
BCL2: B-cell Lymphoma 2
CASP3: Caspase 3
CCND1: Cyclin D1
CRF: Corticotropin-Releasing Factor
DNMT3 L: DNA Methyltransferase 3 Like
E2: Estradiol
EGF: Epidermal Growth Factor
EGFR: Epidermal Growth Factor Receptor
FOS: Fos Proto-Oncogene
FSH: Follicle-Stimulating Hormone
FSHR: Follicle-Stimulating Hormone Receptor
GnRH: Gonadotropin-Releasing Hormone
hCG: Human Chorionic Gonadotropin
HIF-1: Hypoxia-Inducible Factor 1
HIF1A: Hypoxia-Inducible Factor 1 Alpha
HPG: Hypothalamus-Pituitary-Gonadal
IL6: Interleukin 6
JUN: Jun Proto-Oncogene
Ki67: MKI67 (marker of proliferation Kiel 67)
LH: Luteinizing Hormone
LHR: Luteinizing Hormone Receptor
MAPK: Mitogen-Activated Protein Kinase
MMP9: Matrix Metalloproteinase 9
NF-κB: Nuclear Factor Kappa-B
Nrf2: Nuclear Factor Erythroid 2-Related Factor 2
OCCLUDIN: Occludin
OPG: Osteoprotegerin
PCOS: Polycystic Ovary Syndrome
PI3K/AKT: Phosphatidylinositol 3-Kinase/Akt
POI: Premature Ovarian Insufficiency
RANKL: Receptor Activator of Nuclear Factor-κB Ligand
RSA: Recurrent Spontaneous Abortion
SCF/c-kit–PI3K–Bcl-2: Stem Cell Factor/c-kit–Phosphatidylinositol 3-Kinase–Bcl-2
TCM: Traditional Chinese Medicine
TNF-α: Tumor Necrosis Factor-α
TP53: Tumor Protein 53
TRACP-5b: Tartrate-Resistant Acid Phosphatase 5b
VEGF: Vascular Endothelial Growth Factor
VEGFA: Vascular Endothelial Growth Factor A
β-EP: β-Endorphin
